# A systematic review of complex system interventions designed to increase recovery from depression in primary care

**DOI:** 10.1186/1472-6963-6-88

**Published:** 2006-07-16

**Authors:** Jane Gunn, Justine Diggens, Kelsey Hegarty, Grant Blashki

**Affiliations:** 1 Primary Care Research Unit, Department of General Practice, University of Melbourne 200 Berkeley St, Carlton, Melbourne, Australia; 2 ParaQuad Victoria, 208 Wellington Street. Collingwood 3066. Melbourne, Australia; 3 Institute of Psychiatry, Kings College London, London, UK

## Abstract

**Background:**

Primary care is being encouraged to implement multiprofessional, system level, chronic illness management approaches to depression. We undertook this study to identify and assess the quality of RCTs testing system level depression management interventions in primary care and to determine whether these interventions improve recovery.

**Method:**

Searches of Medline and Cochrane Controlled Register of Trials. 'System level' interventions included: multi-professional approach, enhanced inter-professional communication, scheduled patient follow-up, structured management plan.

**Results:**

11 trials met all inclusion criteria. 10 were undertaken in the USA. Most focussed on antidepressant compliance. Quality of reporting assessed using CONSORT criteria was poor. Eight trials reported an increase in the proportion of patients recovered in favour of the intervention group, yet did not account for attrition rates ranging from 5 to 50%.

**Conclusion:**

System level interventions implemented in the USA with patients willing to take anti-depressant medication leads to a modest increase in recovery from depression. The relevance of these interventions to countries with strong primary care systems requires testing in a randomised controlled trial.

## Background

By 2020, depression is projected to become the second most common cause of loss of disability-adjusted life years in the world [1]. The majority of cases are diagnosed and managed by general practitioners [2]. There is evidence for effectiveness of pharmacological and psychological interventions when tested in efficacy trials in well-controlled settings [3-5]. General practice has been criticized for inadequately recognizing and managing depression, and since the early 1990's there has been an increasing push for primary care to implement chronic illness management and collaborative care models to better manage depression [6]. A number of randomised trials testing these complex interventions for depression management have now been completed and published [6-10]. Policy-makers and clinicians are beginning to implement these models, yet it is not clear to what extent these interventions actually improve remission of depression; and if so, for how long. There have been calls for full remission and functional recovery as the most important goal of treatment [11].

We have identified five relevant reviews published in six papers since 2001 [6-10]. Von Korff's editorial reviewed a selection of depression RCTs and concluded that case management was a key ingredient to achieving a positive outcome, yet did not review quality of trials included. Gilbody et al focussed on identifying and describing the educational and organisational interventions for the management of depression in primary care, yet did not focus on recovery from depression as an outcome, nor on trial quality. Badamgarav and colleagues focussed on management programs for depression care, included non-randomised studies and was not specific to primary care. Bijl et al reviewed trials of disease management programs that included screening, they commented on the 'highly divergent' methodological quality of trials yet did not report a formal assessment of trial quality. Dawson et al undertook a meta-analysis of randomised trials recruiting subjects with major depressive disorder conducted in primary care using remission as a key outcome.

These recent systematic reviews have gathered together published articles of randomised trials aimed at improving the management of depression in primary care, yet they vary in their scope and inclusion criteria from this review. None include information about trial quality and only one presents any data on recovery [7].

We report a systematic review of the randomized trials testing chronic illness management approaches for depression in primary care. We refer to these trials as 'systems trials' throughout the paper. We examine the quality of reporting of the published randomized trials and discuss the relevance of their findings to primary care led health systems.

## Method

We developed inclusion criteria to identify all randomised controlled trials implementing interventions at the 'system' level, aimed at management of depression in adult primary care populations and comparing the new 'system' of care with the existing or 'usual' care. Trials were included only if they used a validated tool to assess participants as depressed at baseline and included a follow-up measure of recovery or remission from depression (or results from which recovery levels could be determined). Clustered and individually randomised trials were included.

Trials were classified as at the 'system level' if they tested interventions that included all of the following:

1. A multi-professional approach to patient care. This required that a general practitioner (GP) or family physician and at least one other health professional (e.g. nurse, psychologist, psychiatrist, pharmacist) were involved with patient care.

2. A structured management plan. In line with introducing an organised approach to patient care 'systems' trials were required to offer practitioners access to evidence based management information. This could be in the form of guidelines or protocols. Interventions could include both pharmacological (e.g. antidepressant medication) and non-pharmacological interventions (e.g. patient screening, patient and provider education, counselling, cognitive behaviour therapy).

3. Scheduled patient follow-ups. A 'systems' approach required interventions to have an organised approach to patient follow-up. We defined this as one or more scheduled telephone or in-person follow-up appointments to provide specific interventions, facilitate treatment adherence, or monitor symptoms or adverse effects.

4. Enhanced inter-professional communication. This required that the intervention introduced mechanisms to facilitate communication between professionals caring for the depressed person. This included team meetings, case-conferences, individual consultation/supervision, shared medical records, patient-specific written or verbal feedback between care-givers and was sometimes referred to as 'collaborative care' in the publications.

As this review focussed on interventions for the general adult primary care population, studies that selected for sub-groups of adult patients with depression (eg, patients with specific co-morbidities, patients from specific cultural backgrounds only, samples of all women/men, post-natal depression, or elderly-only samples) were excluded.

### Literature search

A search of Medline (Ovid, see Table 1) and the Cochrane Central Register of Controlled Trials (CCRCT) was conducted in July 2004 for all relevant English-language publications. Search terms included depression, primary care, general practice/practitioners and family practice/practitioners/physicians. Searches were conducted using each word-stem (e.g. depress*) to ensure all variants of each word were captured in the search. No limit was placed on the year of publication. For the Medline search, the search terms were combined with Cumbers and Wentz's strategy which is specific for identifying randomised controlled trials [12]. The search was repeated using PubMed and no further studies were identified. Titles and abstracts were independently read and reviewed by JG or JD, and short-listed articles were discussed by both researchers to determine eligibility. In addition to this search strategy, hand-searches of reference lists in relevant papers were conducted.

### Data extraction

JD systematically extracted the following data from the papers: authors and year of publication, study setting and location, method of screening and inclusion/exclusion criteria, method and level of randomisation, components of interventions, sample size, attrition rates, follow-up times, recovery outcome measures and recovery results.

JD and JG independently examined each publication to assess the degree to which it was reported in accordance with CONSORT recommendations [13-15] and entered this information into a template designed using CONSORT criteria. Where a trial was reported in multiple publications we examined each publication in detail. Resulting tables were independently cross-checked by KH and GB. Any discrepancies were discussed until consensus was reached. Limitations of each trial were discussed by all authors until consensus was reached.

## Results

**Figure 1:**
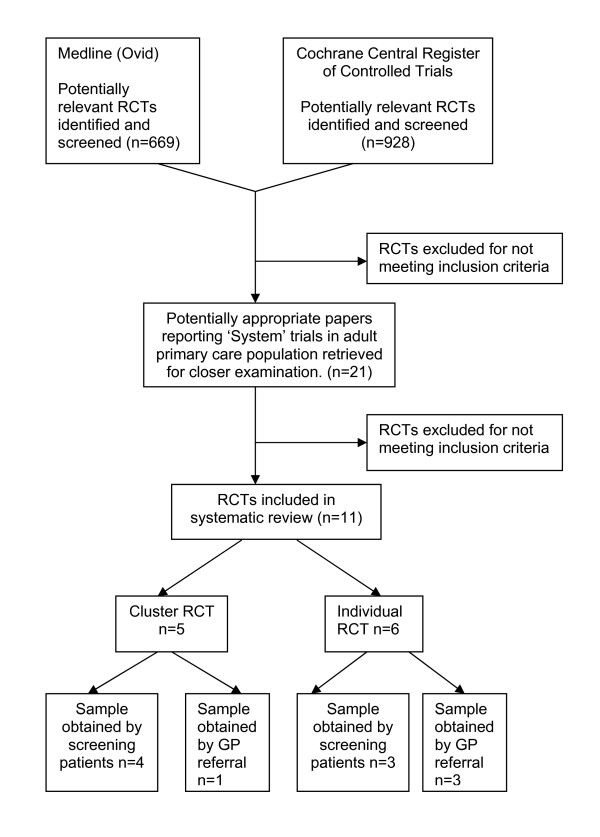
Flow diagram of search for relevant publications.

We identified 928 articles on the CCRTR, and 669 articles on Medline (many trials being identified on both databases, see Figure 1). Eleven trials met all inclusion criteria [16-26]. Trials that were described in multiple publications were considered as a single study and are named in this paper as the first published study.

Table 2 summarises the study location, inclusion criteria, randomisation method and study size and see Additional file 1 which summarises the characteristics of the interventions.

### Representativeness of sample and generalisability of results

Ten of the eleven trials were undertaken in the USA and one in the UK [17]. Three trials [19,21,22], used a practice-based screening approach to identify cases of probable depression whilst the remainder relied upon physician-made referral [16,17,20,23-25] or screening of patients receiving a new antidepressant prescription [18,26]. Details about the number of eligible cases not recruited into studies were not well reported. Where they were reported, issues of generalisability of the trial findings to the population of depressed primary care patients are raised. For example, Rost reports that 16% of those approached refused screening and that 27% of those screened refused a baseline interview [27]. Five of the trials recruited only patients willing to take antidepressant medication [16,18,20,24,26]. The majority of interventions were focussed around improving compliance of patients with antidepressant medication and only two trials specifically included a manualised non-pharmacological intervention [16,21]. All trials were pragmatic trials undertaken in a real world clinical setting.

Table 3. summarises the quality of reporting of trials in accordance with CONSORT criteria (as judged by the authors). No trial was judged as adequately addressing all of the CONSORT criteria. All trials gave good descriptions of the actual interventions delivered. In general the quality of trial reporting when assessed using CONSORT criteria was poor. Of the eleven identified trials five were randomised by cluster and six by individual. The method used to generate the random allocation sequence was reported for seven trials, yet none included a clear description of the method used to implement the random sequence (allocation concealment). Other common omissions were a lack of: clearly stated pre-specified objectives, documented primary and secondary outcomes and planned sub-group analyses, relevant sample size calculations, power to assess recovery and a clear diagram showing participant flow. Many studies inadequately reported attrition rates and even those that did failed to investigate how these rates could have influenced study findings. Only two trials reported any information about attempts to monitor adverse events. Blinding patients to allocation in a randomised trial of a mental health intervention is often impossible, yet few authors discuss the potential biases introduced by the lack of blinding. Allocation concealment and blinding status were poorly reported and no paper presented a discussion of the limitations of lack of blinding. Whilst statistical methods were generally well reported many studies appeared to ignore the problems of multiple testing [28].

Table 4 summarises the follow-up times, attrition rates, measurement tools, blinding and recovery results. Recovery was defined as no longer satisfying criteria for probable depression using the scale included in the study. Some trials reported recovery results as proportions or odds ratios and it was impossible to accurately determine the actual numbers recovered or to independently calculate significance levels. Where actual numbers could be deduced we have included them in the table.

### Meta-analysis

Due to the mix of cluster and individually randomised trials, lack of actual numbers of participants who met recovery criteria being reported, incomplete descriptions of participant flow and variation in: follow-up times, instruments used to measure outcomes; eligibility criteria, severity of depression and co-morbidities, we were unable to confidently utilise quantitative data synthesis techniques.

Trials reported outcomes at varying time-points from three, four, six, 12, 24 to 57 months. It was not always clear why these time-points were chosen. Eight of the trials reviewed showed an increase in the proportion of those recovered in favour of the intervention group (range from 10% to 33%) at the varying follow-up times. Attrition rates ranging from 5% to 50% were reported (see Table 4), yet not taken into account in the reported recovery rates. No trial reported an intention to treat analysis. Four trials reported recovery outcomes at or beyond one year of follow-up [19,21,24,29,30], with three of these trials reporting findings in favour of the intervention [19,21,29,30].

## Discussion

We identified eleven randomised trials testing a system level intervention in primary care and measuring recovery from depression as an outcome. We were able to use the CONSORT criteria and reach agreement about the quality of each trial reported. Overall the quality of reporting was poor. As expected, more recently published trials were more likely to report along CONSORT criteria, yet no trial fully addressed all criteria. Most of the published studies lacked power to measure the effect of the intervention on recovery. Few clearly stated pre-specified objectives and outcome measures. These limitations coupled with the lack of intention to treat analysis and the problematic practice of multiple testing and sub-group analyses makes the interpretation of results and use of meta-analysis techniques problematic.

The trials used a variety of tools to assess depression and recovery and there appeared to be no consensus as to what constitutes a clinically meaningful outcome measure for testing interventions to reduce depression in primary care, nor the best tools to measure it.

### Clinical implications

All but one of the trials reviewed was undertaken in the USA. We know that the primary health care system in the USA is very different from Europe, Canada, Australia and New Zealand. Translating the findings of systems based intervention trials between countries raises interesting challenges for researchers and policy makers; particularly if we acknowledge the complexity in health care[31].

Most of the trials recruited only patients willing to take, or already prescribed, antidepressant medication and all but one used primarily pharmacologically based interventions. The findings from these trials may not be relevant to the broader primary care population who prefer psychological treatment [32]. This is further supported by the work of Bower and Gilbody who report that system level collaborative care interventions tend to be tested on patients with more severe disorders and focus on drug treatment and patients at risk of relapse and recurrence [33]. These findings suggest the need to reconsider the applicability of system level intervention models to those with milder forms of depression.

### Is this review biased?

Our review is biased as we have only included published papers that report recovery data and have judged the trials according to what is recorded in the publication. It should be kept in mind that publication bias tends to favour trials with a positive outcome and it is likely that recovery data is more likely to be reported if it shows in favour of an intervention. We purposefully did not contact authors of the papers included in this review as we wished to assess the evidence as it stands in the public domain. Our review is also limited to English language papers and it is possible that negative trials reported in non-English journals have been excluded.

## Conclusion

System level interventions implemented in the USA, with patients willing to take anti-depressant medication, lead to a modest increase in recovery from depression. Whether or not such systems of care are cost-effective in the long-term is unresolved. The relevance of these interventions to countries that have stronger primary care systems (e.g. UK, Netherlands, Canada, Australia, NZ) is not known. It is inappropriate to assume that these types of interventions can be 'transplanted' to a different health care setting with the same effect as observed in the USA. We require adequately powered randomised trials to test the effectiveness of these models of care in settings outside the USA before widespread implementation occurs.

Outcomes for people experiencing depression are suboptimal [34] and it is almost certain that researchers, policy-makers and clinicians will maintain an interest in re-defining the system of depression care in the community setting [6,35]. It is important that we have high quality randomised trial data to support any major re-engineering of primary care and it appears from our review that the trials testing systems of care for depression managed in the community have suffered from many of the common pitfalls outlined by Chalmers [36].

As a community we need to agree upon the measures to be used when assessing effectiveness of interventions for depression. This is a complicated issue in itself, and Dowrick highlights the need for debate on how we view and measure depression [37]. If we agree that functional recovery and full remission is the goal of management [11] we need to agree upon a consistent way of measuring it.

We hope that this review will assist researchers developing trial protocols for interventions aimed at reducing depression, by encouraging them to think again about: defining the components of their system intervention, planning for a publication that addresses CONSORT reporting criteria, contributing their data to a quantitative meta-analysis and including a cost-effectiveness data analysis.

## Competing interests

The author(s) declare that they have no competing interests.

## Authors' contributions

JG is responsible for conception and design, analysis and interpretation of data. JG drafted manuscript and gave final approval of the version to be published. JD is responsible for analysis and interpretation of the data, drafting of the manuscript tables and contributing to revisions of the manuscript. KH and GB are responsible for checking of data analysis and critically revising the article. All authors gave approval of the version to be published.

## Pre-publication history

The pre-publication history for this paper can be accessed here:



## Supplementary Material

Additional File 1 Table: Characteristics of the interventions used in system intervention trials. Click here for file

## Figures and Tables

**Table 1:** Medline search strategy

001 controlled clinical trials/
002 randomized controlled trials/
003 exp research design/
004 multi-center studies/
005 single-blind method/
006 clinical trial.pt.
007 ((single or double or treble or triple) adj5 (mask$ or blind)).tw.
008 placebos/or placebo$.tw.
009 or/1–8
010 depress$.mp. [mp = title, original title, abstract, name of substance word, subject heading word]
011 (family practi$ or general practi$ or primary care$ or family physician$).mp. [mp = title, original title, abstract, name of substance word, subject heading word]
012 9 and 10 and 11

**Table 2:** Study location, inclusion criteria, method of randomization and sample size.

**First Author and Year**	**Study location, participant inclusion criteria, method of randomization and sample size**.
Katon 1996 [16]	• Puget Sound, USA • Adults (18–80 yrs) considered by the GP as having "definite or probable major depression" were referred to the study over a one-year period and screened using the Symptom Checklist (SCL-20). Participants scoring >= .75 and who were willing to take anti-depressant medication were recruited into the study. Some exclusion criteria were applied. 74.1% were female. • Stratified (by SCL-20) with randomization of individual patients in blocks. • Usual Care (UC) n = 76 vs Collaborative Care Intervention (I) n = 77.
Mann 1998. [17]	• UK-wide. • Adults (18–74 yrs) considered by the GP to have had depression for at least 4 weeks (regardless of prior treatment) were recruited into the study over a 2.5 year period (no further screening was undertaken). Some exclusion criteria were applied. 78% were female. • Simple randomization of individual patients. • Usual Care (UC) n = 148 vs Feedback & Nurse Monitoring Intervention (I) n = 271. • Note – Two interventions were conducted. Only the one meeting criteria for a system intervention is included here.
Katon 1999. [18]	• Puget Sound, USA. • Adults (18–80 yrs) receiving a new anti-depressant prescription (i.e., no prescription in past 120 days) for anxiety or depression were identified by clinic databases. Screening was conducted 6–8 wks later to select for those with persistent symptoms of depression (as defined by Structured Clinical Interview for DSM-III-R (SCID) > 4 symptoms of depression, and Symptom Checklist (SCL-20) >1, or SCID<4 symptoms of depression but SCL-20>1.5). Some exclusion criteria were applied. 74.5% were female. • Stratified (by SCL-20) randomization of individual patients in blocks. • Usual Care (UC) n = 114 vs Stepped Collaborative Care Intervention (I) n = 114.
Katzelnick 2000. [19]	• Wisonsin, Washington and Massachusetts, USA. • Adults (25–63 yrs) who were "high utilizers" of health clinic (ie, frequency of ambulatory visits above the 85^th ^percentile) were identified on clinic databases. Eligible participants were screened for major depression or major depression in partial remission using the Structured Clinical Interview for DSM-IV (SCID). Patients meeting second-stage screening criteria on Hamilton Depression Rating Scale (Ham-D; scores = >15) were enrolled. Participants were not on anti-depressants at baseline. Some exclusion criteria were applied. 77% were female • Cluster randomization by practice. • Usual Care (UC) n = 189 vs Depression Management Program (I) n = 218.
Simon 2000. [20]	• Puget Sound, USA • Adults (age range not reported) receiving a new prescription for anti-depressants (i.e., no prescription in past 120 days) were identified by clinic databases and recruited into the study. No further screening was undertaken. Some exclusion criteria were applied. Aprox. 72% were female. • Stratified (by clinic) with randomization of individual patients. • Usual Care (UC) n = 196 vs Feedback and Care Management Intervention (I) n = 196. • Note – Two interventions were conducted. Only the one meeting criteria for a system intervention and is included here.
Wells 2000 [21,29,39]	• 7 regions in the USA. • Consecutive adults (18+yrs) attending clinics over a 5–7 month period were screened for probable or persistent depression using the Composite International Diagnostic Interview (CIDI – 2 weeks of depressed symptoms or probable depression in the past year, with at least one week of depression in the past month). Some exclusion criteria were applied. 71% were female. 30% of participants were Hispanic (deliberate choice of practices to oversample for Mexican Americans). • Cluster randomisation (by practice), matched in blocks of 3 on patient demographics, clinician specialty and distance to mental health providers. Stratification by proportion of Mexican American patients occurred in one region only. • Usual Care (UC) n = 443 vs Quality Improvement-Therapy (I-Therapy) n = 489 vs Quality Improvement-Medication (I-Meds) n = 424.
Rost 2000 [22,27,30,40,41]	• Clinics across the USA. • Consecutive adults (18+yrs) attending clinics for routine-length visits were screened over an 18 month period for "probable major depression" on the WHO-Composite International Diagnostic Interview (CIDI – 2 weeks of depressed symptoms or probable depression in the past year, with at least one week of depression in the past month). Those meeting second-stage screening criteria on the Inventory to Diagnose Depression (IDD >5 of 9 depression symptoms in previous 2 weeks) were enrolled. Some exclusion criteria were applied. 84% were female. • Cluster randomization (by practice), matched in blocks (metro vs rural practices) on proportion of patients receiving guideline-concordant care. • Usual Care (UC) n = 240 vs Enhanced Care Intervention (I) n = 239 • Recovery was only reported for sub-groups of Rost's 2001 sample, in later publications. • Smith 02 – Exclusion of n = 96 elderly (65+) from Rost 2001 sample. 81% were female. Usual Care (UC) n = 195 (insured n = 150, uninsured n = 45) vs Enhanced Care Intervention (I) n = 188 (insured n = 140, uninsured n = 48). • Rost 2002 – Exclusion of n = 268 "treatment resistant" participants from Rost 2001 sample. 84% were female. Usual Care (UC) n = 96 vs Enhanced Care Intervention (I) n = 115
Datto 2003 [23]	• Pennsylvania, USA • Adults (age range not reported) with "symptoms suggestive of depression" were identified by the GP and recruited into the study (no further screening was undertaken). Some exclusion criteria were applied. 60.7% were female. • Cluster randomisation (by practice). • Usual Care (UC) n = 31 vs Telephone Disease Management Intervention (I) n = 30.
Finley 2003 [26]	• California, USA. • Adults (age range not reported) were referred to the study by their primary care provider (GP) when starting new antidepressant medication (ie, no medication taken in past 6 months) for depression (no further screening was undertaken). Some exclusion criteria were applied. Patients were paid $20 at end of study. 85% were female • Simple randomization of individual patients. • Usual Care (UC) n = 50 vs Collaborative Care Intervention (I) n = 75.
Capoccia 2004 [24,42],	• Seattle, USA (academic clinic) • Adults (18+) with a newly diagnosed depression episode and anti-depressant prescription (as determined by their health care provider) were referred to the study, then screened for depression using PRIME-MD (cut off criteria not reported). Some exclusion criteria were applied. 57% were female. • Simple randomization of individual patients. • Usual Care (UC) n = 33 vs Enhanced Care Intervention (I) n = 41
Dietrich 2004 [25] [43]	• Clinics across the USA • Adults 18+ who were commencing or changing treatment for depression were identified by clinicians and referred for a structured interview. Those with DSM-IV major depression or dysthymia, and with Hopkins Symptom Checklist-20 >= 0.5 were eligible. Participants had to be willing to take anti-depressant treatment or be referred for psychological counselling. Some exclusion criteria were applied. 80% were female. • Cluster randomization (by practice), stratified by health care organisation, and matched by GP specialty, presence of clinic mental health care and distance from the organisations central office. • Usual Care (n = 181) vs Quality Improvement Intervention (I) n = 224

**Table 3:** The quality of reporting of trials^i ^in accordance with CONSORT criteria [14,15] [13]

Consort item #	Text in *italics *= consort criteria relevant to *cluster *randomised trials only.	Trials that reported information as outlined by CONSORT^ii ^**Cluster RCTs are in bold**.
1. Design	How participants were allocated to interventions (eg "random allocation", "randomised", or "randomly assigned"), *specifying that allocation was based on clusters*.	A, B, C, E, **G, H**, J, K, **L**
2. Background	Scientific background and explanation of rationale, *including rationale for using a cluster design*.	A, B, C, E, **G, H**, J, K
3. Participants	Eligibility criteria for participants *and clusters *and the settings and locations where data were collected.	A, B, C, E, **G, H**, J, K, **L**
4. Interventions	Precise details of the interventions intended for each group, *whether they pertain to the individual level, the cluster level, or both*, **a**nd how and when they were actually administered.	A, B C, **D**, E, **F, G, H, I**, J, K, **L**
5. Objectives	Specific objectives and hypotheses *and whether they pertain to the individual level, the cluster level, or both*.	A, B C, **D**, E, **F, G, H, I**, J, K, **L**
6. Outcomes	Clearly defined primary and secondary outcome measures, *and whether they pertain to the individual level, the cluster level, or both*.	B, **F, G, H**, K, **L**
	And, when applicable, any methods used to enhance the quality of measurements (eg, multiple observations, training of assessors)	Not assessed/judged
7a. Sample size	How *total *sample size was determined *including method of calculation, number of clusters, cluster size, a coefficient of intracluster correlation (ICC or k), and an indication of its uncertainty)*.	B, E, J, K, **L**
7b. Sample size	And, when applicable, calculation of interim analyses and stopping rules.	K (no other article calculated interim analyses)
8. Sequence generation	Method used to generate the random allocation sequence, including details of any restriction, (eg blocking, stratification, *matching*).	A, B, C, E, **F, H**, K, **L**
9. Allocation concealment	Method used to implement the random sequence (eg, numbered containers or central telephone), *specifying that allocation was based on cluster rather than individuals and *clarifying whether the sequence was concealed until interventions were assigned.	None
10. Implementation	Who generated the allocation sequence, who enrolled participants **a**nd who assigned participants to their groups.	**G, H**
11a. Blinding	Whether or not participants, those administering the interventions and those assessing the outcomes were blinded to group assignment.	J, K
11b. Blinding	If done, how the success of blinding was evaluated.	F **(no others assessed blinding)**
12a. Statistical methods	Statistical methods used to compare groups for primary outcome(s), *indicating how clustering was taken into account*.	**A, B, C**, D, **E**, F, G, H, **J, K**, L
12b. Statistical methods^iii^	Methods for additional analyses, such as subgroup analyses and adjusted analyses	**F**
13. Participant flow^iv^	Flow of *clusters and *individual participants through each stage. Specifically for each group report the numbers of clusters and participants randomly assigned, receiving intended treatment, completing the study protocol and analysed for the primary outcome.	B, **D, F**, **H **(see footnote for further explanation)
	Describe protocol deviations from study as planned, together with reasons.	Not assessed/judged
14. Recruitment	Dates defining the periods of recruitment and follow-up	B, **F, G, H**, K, **L**
15. Baseline data	Baseline demographic and clinical characteristics *for the individual and cluster levels as applicable*.	A, B C, E, **G, H, I**, J, K, **L**
16. Numbers analysed^v^	Number of *clusters and *participants (denominator) in each group included in [recovery] analyses and whether analysis [not specific to recovery] was by "intention to treat". State the results in absolute numbers when feasible (eg, 10/20 not 50%)	B, **D**, **F**, J, **L**
7. Outcomes and estimation^v^	For [recovery analyses], a summary of results for each group *for the individual or cluster level as applicable *and the estimated effect size and its precision (eg, 95% CI)".	**None**
18. Ancillary analyses	Address multiplicity by reporting any other analyses performed, including subgroup analyses and adjusted analyses, indicating those prespecified and those exploratory.	None (N/A for L)
19. Adverse events	All important adverse events or side effects in each intervention group.	None
20. Interpretation	Interpretation of the results, taking into account study hypotheses, sources of potential bias or imprecision and the dangers associated with multiplicity of analyses and outcomes.	None
21. Generalisability	Generalisability (external validity) *to individuals and/or clusters (as relevant) *of the trial findings.	A, C, **D**, E, **F, G, H, I**, J, K, **L**
22. Overall evidence	General interpretation of results in the context of current evidence	A, B, **D**, E, **F, G, H, I**, J, K, **L**

i A = Katon 1996, B = Mann, 1998, C = Katon 1999, D = Katzelnick, 2000, E = Simon 2000, F = Wells, 2000 (and Wells 1999), G = Smith 02 (and Smith 00, 01, Rost 00 and 01), H = Rost 2002 (and Rost 00), I = Datto 03, 2002, J = Finley 03, K = Capoccia 04 (and Boudreau 02), L = Dietrich 04a (and Dietrich 04b, and web appendices).

^ii ^*For publications to be considered as reporting information in accordance with CONSORT criteria, publications had to provide an explicit statement or clear and unambiguous information outlining details relevant to that CONSORT criteria. As many CONSORT items can be broken down into multiple components, each publication was only considered to have met CONSORT criteria if *all *components were adequately addressed.

*If a publication referenced another article that included the required information (such as when there were multiple publications about a single trial), this article was also used in judging CONSORT criteria where indicated.

^iii ^For studies A, G, H, I, K, L, this item was either Not Applicable (because they did not report additional analyse or subgroup analyses), or was too difficult to judge as they described statistical methods but did not clearly specify the outcomes they were used for (B, C, D, E).

^iv ^When coding this item, the number analysed for primary outcome was difficult to judge as most articles reported multiple (primary) outcomes.

^v ^For simplicity, items 16 and 17 were coded as they relate to recovery data only.

**Table 4:** Recovery results of system intervention trials.

**1^st ^Author and Date**	**Follow-up Times and Attrition**	**Measures of Recovery and Recovery Results**
Katon 1996 & Lin 1999.	• F/U at 1, 4, 7 & 19 mths. • Attrition^# ^at 4 mths: UC = 14/76* (18.4%*), I = 11/77* (14.3%*) • RA's completing F/U were blind to intervention status.	Recovery = Proportion with 1 or less symptoms of depression on the Inventory for Depressive Symptomatology (IDS). Recovery was only reported for sub-groups in the article (people with Major depression vs Minor depression). Usual Care and Intervention data were deduced from sub-group data provided. • At 4 mths: UC = 35/62* (56%*), I = 45/66* (68%*). No statistical comparisons were provided.
Mann 1998.	• F/U at 4 mths. • Attrition: UC = 14/148 (9.5%), I = 20/271 (7.4%) • RA's completing F/U were not blind to intervention status.	Recovery = Change in proportion not meeting DSM-III criteria for major depression on the Nurse Assessment Interview (NAI) from baseline to follow-up. • At 4 mths: UC = 79/134* (59%*), I = 123/251* (49%*), ns.
Katon 1999.	• F/U at 1, 3 & 6 mths. • Attrition at 3 mths: UC = 17/114* (14.9%*), I = 18/114* (15.8%*) • Attrition at 6 mths: UC = 17/114* (14.9%*), I = 19/114* (16.6%*) • RA's completing F/U were blind to intervention status.	Recovery = Proportion with 1 or 0 symptoms of depression on the Structured Clinical Interview (SCID) for DSM-IV. • At 3 months: UC = 22/97* (23%), I = 38/96* (40%), p = .01 • At 6 months: UC = 30/97* (31%), I = 42/95* (44%), p = .05
Katzelnick 2000.	• F/U at 6 wks, 3, 6 & 12 mths. • Attrition at 12 mths: UC = 12/189 (6.3%), I = 15/218 (6.9%). • RA's completing F/U were blind to intervention status.	Recovery = Proportion with Hamilton Depression Rating Scale (HDRS) < 7. • At 12 mths: UC = 49/177 (27.7%), I = 92/203 (45.3%), p < .001 • At 12 mths: Number to Treat = 5:1
Simon 2000.	• F/U at 3 & 6 mths. • Attrition not reported. • RA's completing F/U were blind to intervention status.	Recovery = The inverse of odds ratios for meeting criteria for major depression on the Structured Clinical Interview (SCID) for DSM-IV. • Across follow-ups, the Odds Ratio = 0.45 (0.24–0.86) indicates the probability of recovery was significantly higher in the Intervention group
Wells 2000 & Sherbourne 2001 & Wells 2004.	• F/U at 6, 12, 24 & 57 mths. • Attrition at 6 mths: UC = 57/443 (12.9%), I = 143/913 (15.7%). • Attrition at 12 mths: UC = 69/443 (15.6%), I = 161/913 (17.6%). • Attrition at 24 mths (estim.): UC = 65/443* (14.7%), I-Meds = 62/424* (14.7%), I-Therapy = 72/489* (14.7%) • Attrition at 57 mths: UC = 131/443 (29.6%), I-Meds = 102/424 (24.0%), I-Therapy = 132/489 (27%) • RA's completing F/U were not blind to intervention status.	Recovery = Proportion no longer meeting probable or persistent depression on the Composite International Diagnostic Interview (CIDI). • At 6 mths, UC = 193/386* (50.1%*), I-Therapy & I-Meds combined = 463/770* (60.1%*), p = .005 • At 12 mths: UC = 183/374* (48.8*)%, I-Therapy & I-Meds combined = 439/752* (58.4%*), p = .04 • At 24 mths: UC = 223/338* (66%*) vs I-Meds = 221/362* (61%*), ns. UC = 223/338* (66%*) vs I-Therapy = 337/489* (69%*), ns • At 57 mths: UC = 176/312* (56.4%*) vs I-Meds = 200/322* (62.1%*), ns. UC = 176/312* (56.4%*) vs I-Therapy = 228/357* (63.8%*), p = .05 Recovery = Proportion with Center for Epidemiologic Studies – Depression (CES-D, Modified version) < 20. • At 6 mths: UC = 137/386* (35.6%), I-Therapy & I-Meds combined = 343/770* (44.6%*), p = .005 • At 12 mths: UC = 144/374* (38.6%), I-Therapy & I-Meds combined = 342/752* (45.5%*), p = .04
Rost 2001 & Smith 2002 & Rost 2002	• F/U at 6, 12, 18 & 24 mths. • N = 479 were recruited to original trial (Rost 00). Smith 2002 and Rost 2002 are sub-group follow-ups. • Smith 02: Attrition at 24 mths: UC = 48/195* (24.6%*), I = 70/188 (36.7%*). • Rost 02: Attrition at 24 mths: UC = 13/96 (13.5%), I = 46/115 (40%) • RA's completing F/U were blind to intervention status.	Smith 2002 Recovery = Proportion not meeting Composite International Diagnostic Interview (CIDI) criteria for major depression. Recovery was only reported for sub-groups in this article (uninsured vs insured, no significant difference was found). Usual Care and Intervention data were deduced from sub-group data provided. • At 24 mths: UC = 113/148* (76.4%*), I = 92.5/119* (78%*). Rost 2002 Recovery = Proportion of participants with Centre for Epidemiological Studies-Depression (CES-D) <16. • At 24 mths: UC = 34/83* (41%) vs I = 51/69* (74%), p = .02
Datto 2003	• F/U at 16 weeks. • Attrition at 16 weeks: UC = 5/31* (16.1%) vs I = 6/30* (20%) • Unclear if nurses completing F/U were blind to intervention status.	Recovery = Proportion of participants with Centre for Epidemiological Studies-Depression (CES-D) <11. • At 16 weeks: Odds Ratio = 3.48 (0.85–14.29), n.s (p = .08) Recovery = Proportion of participants with CES-D<16 • At 16 weeks: Odds ratios = 6.58 (1.57–27.03), significant (p = .01) in favour of intervention Recovery = Proportion of participants not meeting Mini-International Neuropsychiatric Interview (MINI) criteria for major depression. • At 16 weeks: Odds ratio = 0.52 (0.13–2.03), n.s, p = .66
Finley 2003	• F/U at 6 mths • Attrition: UC = 25/50 (50%) vs I = 16/75 (21%) • F/U measures were completed by postal self-report questionnaires.	Recovery = Proportion of participants with Brief Inventory for Depressive Symptoms (BIDS) < 9 • At 6 mths UC = 14.6/25* (58.3%), I = 32.8/59* (55.6%), ns (p = .36)
Capoccia 2004	• F/U at 3, 6, 9 and 12 months. • Attrition by Total N: At 3 mths = 3/74 (4%), At 6 & 9 mths = 4/74 (5%), At 12 mths = 5/74 (7%) (attrition was approximately equal in both groups) • RA completing F/U was blind to intervention status.	Recovery = Proportion not meeting criteria for major depression on the Structured Clinical Interview (SCID) for DSM-IV and Symptom Checklist (SCL-20). Exact criteria/cut-offs were not specified. • % with major depression did not differ significantly @ 3, 6, 9, 12 mths, p = .32. • At 12 mths: Recovery deduced from data in article as: UC = 31/31* (100%*), I = 37/38* (97%*)
Dietrich 2004	• F/U at 3 & 6 months. • Attrition at 3 mths: UC = 29/181 (16%) vs I = 39/224 (17%) • Attrition at 6 mths: UC = 35/181 (19%) vs = 45/224 (20%) • Interviewers completing F/U were blind to intervention status.	Remission = Proportion with Symptom Checklist (SCL-20) < 0.5. • At 3 mths: UC = 25/152 (16.5%), I = 48/183 (26.2%), p = .018 • At 6 mths: UC = 39/146 (26.7%), I = 66/177 (37.3), p = .014

* Figures (eg, n, percentages) were deduced from data in the article where indicated by an asterix. # Attrition rates were sometimes only reported for total subjects, rather than by arm of the trial.
