# Supplementary material for: A systematic review of complex system interventions designed to increase recovery from depression in primary care
Source: BMC Health Serv Res. 2006 Jul 16;6:88. doi: 10.1186/1472-6963-6-88 (PMC1559684; doi:10.1186/1472-6963-6-88)
Supplement: Additional File 1 — Table: Characteristics of the interventions used in system intervention trials. [file 1472-6963-6-88-S1.pdf]

Characteristics of the interventions used in system intervention trials

First Author and Year	Characteristics of the Intervention
Katon 1996 [16]	UC - GP education (brief seminar on medication and behavioural treatment for depression). Most patients were prescribed anti-depressant medications, with 2-3 follow-up visits over 3 months.<br><br>Intervention<br>• Multiprofessional Approach = GP, Psychologist and Psychiatrist.<br>• Structured Management Plan = Physician education (as in UC) and patient education. Most patients were prescribed anti-depressant medications. Patients received manualised cognitive behavioural therapy for depression, as well as counselling around medication adherence by Psychologist. Weekly psychiatrist review of patient progress (medication adherence and side-effects) at team meetings. Optional referral to psychiatrist for non-responders.<br>• Scheduled Patient Follow-Up = 4-6 visits with Psychologist over 6 wks & 4 telephone contacts over 24 wks.<br>• Enhanced Professional Communication = Psychologist provided written feedback to GP after each patient contact. Weekly GP feedback from reviewing Psychiatrist. GP case-consultation with study psychologists on an as need basis.
Mann 1998. [17]	UC : Baseline psychiatric assessment completed by nurse.<br><br>Intervention<br>• Multiprofessional Approach = GP and Nurse.<br>• Structured Management Plan = As in the UC, plus manualised in-person monitoring of mental state, medicine compliance, education and social intervention by the study Nurse.<br>• Scheduled Patient Follow-Up = 8 hours of nurse contact with each patient over 4 months.<br>• Enhanced Professional Communication = “Ongoing” feedback between Nurse and GP, including results of baseline assessments.
Katon 1999. [18]	UC = Medication, monitoring and referral to mental health provider if necessary.<br><br>Intervention<br>• Multiprofessional Approach = GP, Psychiatrist and optional involvement of Psychologist.<br>• Structured Management Plan = Patient education. Patient visits to psychiatrist regarding anti-depressant medications. Monthly monitoring (by psychiatrist) of medication via automated pharmacy data. Specialty consultation between GP, psychiatrist and patient in cases of severe medication side-effect or non-response to treatment. Optional referral to Psychologist.<br>• Scheduled Patient Follow-Up = Two initial in-person consultations with psychiatrist, with brief telephone contact between visits. Additional consultations provided if necessary for up to 3 months.<br>• Enhanced Professional Communication = Psychiatrist feedback to GP after each patient contact and monthly feedback of cases of premature discontinuation.
Katzelnick 2000. [19]	UC = Patients were told their screening suggested depression, and were encouraged to consult with their GP.<br><br>Intervention<br>• Multiprofessional Approach = GP, Study Coordinators and Psychiatrist.<br>• Structured Management Plan = Patient education, physician education (on assessment and pharmacotherapy). Scheduled F/U’s made with GP. Initiation of anti-depressant medication by GP (when not contraindicated) according to a pre-specified algorithm. Regular telephone contact between Study Coordinators and patients to monitor prescription refills. Referral to psychiatrist when medication not successful or when treatment complicated.<br>• Scheduled Patient Follow-Up = GP visits at 1, 3, 6, & 10 weeks, then every 10 weeks. Study coordinators contact with patients at 2, 10 and 18, 32 and 40 weeks (if need)<br>• Enhanced Professional Communication = Study Coordinator feedback to GP regarding medication adherence, response and adverse effects after each patient contact. Periodic case reviews and telephone consultation between GP and consulting psychiatrist.
Simon 2000. [20]	UC=Standard services provided. No further description.<br><br>Intervention<br>• Multiprofessional Approach = GP, Care Manager and Psychiatrist.<br>• Structured Management Plan = Care Manager telephone contact (twice) with patient to assess current anti-depressant use, side-effects and severity of depression symptoms, and provide encouragement and support. Care Managers also assisted in the GPs carrying out their patient management plans.<br>• Scheduled Patient Follow-Up = Care Manager telephone contact with patients at 8 & 16 wks<br>• Enhanced Professional Communication Care Manager feedback and recommendations to GP after patient

	contact. Weekly supervision of Care Managers by Psychiatrist.
Wells 2000<br><br>[21, 29, 38]	UC = Clinical Medical Directors received written guidelines on depression.<br>Intervention<br>• <u>Multiprofessional Approach</u> = GP, Nurse, Psychiatrist, Psychologist and Local Leaders.<br>• <u>Structured Management Plan</u> = Training of expert “local leaders” in each clinic (a GP, Nurse Supervisor and Psychologist/Psychiatrist). Initial and ongoing local leader training, support and supervision of clinic GP’s, Nurses, and Psychologists (through distribution of clinical manuals, lectures, academic detailing, supporting materials and supervision) in discipline-relevant treatment and collaborative care.<br>• All Participants received initial Nurse appointment to assess, educate and discus treatment options. Nurse feedback provided to GP to assist treatment planning. Patient choice of treatment modality; either Medication (I-Med group) or Therapy (I-Therapy group).<br>• I-Meds – Ongoing Nurse contact with patient to assess and encourage medication adherence, with written feedback provided to GP and Psychiatrist. Patients visited their GP as needed, with the option of a specialty psychiatrist consultation. Participants could access practice Therapists (but not study Therapists).<br>• I-Therapy –Psychologists provided manualised group or individual Cognitive Behavioural Therapy to patients. Patients were not prevented from receiving medication.<br>• <u>Scheduled Patient Follow-Up</u> = All patients visited Nurse at 2wks. Patients in I-Meds received bimonthly then monthly Nurse contact with patient (for 6 or 12 months depending on randomisation). Patients in I-Therapy received 12-16 manualised CBT sessions (or 4 if depression was minor).<br>• <u>Enhanced Professional Communication</u> Local leaders supplied materials to facilitate communication between staff. Regular feedback to GP from Nurses and Psychologists in each intervention. Supervision provided to Nurses and Psychologists by local leaders. Monthly assessment and feedback about intervention provided to clinic staff by local leaders at team meetings. Local leaders provided periodic supervision for Nurses and Psychologists.
Rost 2000<br><br>[22, 27, 30, 39, 40]	UC=GPs were not systematically informed of screening results.<br><br>Intervention<br>• <u>Multiprofessional Approach</u> = GP, Nurse, Psychiatrist, and other clinic staff.<br>• <u>Structured Management Plan</u> = All medical and non-medical staff received training in depression treatment guidelines. An initial GP and Nurse appointment was scheduled, then ongoing Nurse telephone or in-person F/U and care management was provided (assessment, education, monitoring of symptoms). Nurses assisted patients to access treatment (eg, source funding according to patients’ choice of medication or psychotherapy), facilitated referrals, and addressed barriers to care (eg, funding) in coordination with GP and Psychiatrist.<br>• <u>Scheduled Patient Follow-Up</u> = Nurse Care Management was provided weekly for 5 to 7 weeks. At 6 months, follow-ups continued on a monthly or 3-monthly basis (depending on need) until end of two year trial.<br>• <u>Enhanced Professional Communication</u> = Monthly nurse feedback (with recommendations reviewed by psychiatrist) provided to GP throughout study.
Datto 2003<br><br>[23]	UC= Disease Management (DM) specialists (nurse, social worker or psychologist) completed baseline assessment, but no further involvement.<br><br>Intervention<br>• <u>Multiprofessional Approach</u> = GP (family practice or internal medicine), Disease Management (DM) specialists (nurse, social worker or psychologist) and Psychiatrist.<br>• <u>Structured Management Plan</u> = Initial DM assessment with ongoing telephone F/U to assess depression symptoms, provide information and education, and encourage adherence of clinician recommendations.<br>• <u>Scheduled Patient Follow-Up</u> = Initial patient assessment with F/U every 3 weeks (for 16 weeks)<br>• <u>Enhanced Professional Communication</u> = Written feedback (symptom severity and adherence information) provided to GPs after each assessment. GPs could consult DM psychiatrist on an as need basis. DM specialists received weekly supervision with a psychiatrist.
Finley 2003<br><br>[24]	UC=GPs were notified of study participants and care was provided as usual.<br><br>Intervention<br>• <u>Multiprofessional Approach</u> = GP, Clinical Pharmacist and Psychiatrist.<br>• <u>Structured Management Plan</u> = Pharmacists conducted intake interview with patient (to take history and asses severity of symptoms) and provided guideline-based medication. Pharmacists provided scheduled telephone F/Us with patients to monitor adherence, treatment response and adverse effects, and provide ongoing patient education about depression, medication and treatment options.<br>• <u>Scheduled Patient Follow-Up</u> = In-person Pharmacist visits were scheduled at baseline, 6 and 24 weeks while telephone F/Us with Pharmacist were at 1,2, 4, 10 and 6 weeks.<br>• <u>Enhanced Professional Communication</u> = Pharmacists entered notes into the patients’ medical records at each contact. GP / pharmacist communication occurred when changes in medication were recommended, and a written treatment summary was provided to GP at 24 weeks. Pharmacists received weekly

	supervision and as-needed consultation from a psychiatrist.
Capoccia 2004<br><br>[25, 41],	UC=GPs were prompted to use all resources available to them in their clinic.<br><br>Intervention<br>• Multiprofessional Approach = GP, Clinical Pharmacist and Psychiatrist<br>• Structured Management Plan =.Pharmacists provided regular telephone calls to provide patients support and education, and review of medication type, timing, dosage and adverse effects. Medication for related problems was also discussed (eg, sexual dysfunction, insomnia). In-person patient-visits with GP were encouraged at two time points. Patients with suicidal ideation, and non-responders by weeks 8-10 were referred to psychiatrist.<br>• Scheduled Patient Follow-Up = Pharmacist calls were scheduled at 1, 2, 3, 4, 6, 8, 10, 12 weeks then every second month until 12 months. Scheduled GP appointments were encouraged at week 4 and 12.<br>• Enhanced Professional Communication = All patient contacts were recorded in patient file. Informal bimonthly case reviews were held between Pharmacist and Psychiatrist.
Dietrich 2004<br><br>[26] [42]	UC= GP education on the treatment of depression, and staff training in study procedures.<br><br>Intervention<br>• Multiprofessional Approach = GP, Psychiatrist, Care Manager (person with primary care or mental health background) and clinic staff.<br>• Structured Management Plan =Training on the treatment of depression and study procedures provided to all staff/professionals involved. GP treatment (including diagnostic evaluation, management and F/U) was provided to patients, with ongoing telephone support from Care Mangers (to assist with “self management” strategies (eg, exercise) and overcoming barriers to the clinician’s treatment plan). The PHQ-9 was administered each call, to assist in diagnosis and treatment monitoring.<br>• Scheduled Patient Follow-Up = GPs were encouraged to schedule at least 3 F/U appointments. Care Manager support was provided monthly until remission.<br>• Enhanced Professional Communication = Care Managers provided written feedback to GP each month, with psychiatrist providing treatment recommendations as well (to care manger or clinician directly). Psychiatrist available to provided telephone advice to GP. Weekly telephone supervision of Care Mangers by Psychiatrist.
